# Highly selective toxic and proapoptotic effects of two dimeric ribonucleases on thyroid cancer cells compared to the effects of doxorubicin

**DOI:** 10.1038/sj.bjc.6601491

**Published:** 2004-01-06

**Authors:** D Spalletti-Cernia, R Sorrentino, S Di Gaetano, R Piccoli, M Santoro, G D'Alessio, P Laccetti, G Vecchio

**Affiliations:** 1 Istituto di Endocrinologia ed Oncologia Sperimentale del CNR c/o Dipartimento di Biologia e Patologia Cellulare e Molecolare, Università di Napoli Federico II, via S. Pansini 5, 80131 Naples, Italy; 2 Dipartimento di Chimica Biologica, Università di Napoli Federico II, Naples, Italy

**Keywords:** cytotoxic RNases, cell malignancy, apoptosis, thyroid cells and tumours

## Abstract

The lack of selectivity of conventional antitumour drugs against cancer cells is responsible for their high toxicity. The development of new tumour-specific drugs is therefore highly needed. We tested the cytotoxic effects and the nature of cell death induced by a naturally dimeric bovine RNase and a newly engineered dimeric human RNase upon three genetically well-defined normal and malignant thyroid cell systems. RNases effects were compared with those of doxorubicin, a conventional antineoplastic drug. Our results show significant and selective proapoptotic effects exerted on tumour cells by both RNases, the strength of their cytotoxic and apoptotic activity being directly related to the degree of cell malignancy. No toxic effects were observed upon normal cells. Doxorubicin showed, instead, cytotoxic and apoptotic effects also against normal cells. The *in vitro* results were corroborated by the antitumour action of both dimeric RNases towards a malignant human thyroid tumour grown in nude mice. These results indicate a selective action of dimeric RNases against cancer cells and suggest the potential application of these molecules or their derivatives to the treatment of aggressive subtypes of thyroid cancer.

Most of the known conventional antineoplastic drugs lack specificity against cancer cells, exerting instead unfavourable reactions in cancer patients due to their toxic effects on normal tissues (Hurley, 2002). Recent advances in cancer molecular biology allowed the discovery of discrete molecular lesions in key proteins responsible for the development of a fully aggressive cancer phenotype (Chabner *et al*, 1998; Gibbs, 2000). These advances have in turn led to the development of new anticancer drugs possessing selective activity towards tumour cells bearing those specific genetic lesions (Gibbs, 2000; Bange *et al*, 2001). By this approach, a successful treatment can be achieved only in those types of tumours bearing the genetic lesion specifically targeted by the drug.

Some ribonucleases are endowed with a strong antineoplastic activity. The best known members of this family are onconase, a protein extracted from *Rana pipiens* and currently tested in phase II and III clinical trials (Wu *et al*, 1993; Juan *et al*, 1998; Halicka *et al*, 2000,^2002^; Lee *et al*, 2000; Mikulski *et al*, 2002; Saxena *et al*, 2002), and seminal ribonuclease, extracted from bull semen (BS-RNase) (Youle and D'Alessio, 1997). BS-RNase, the only dimeric member of the pancreatic-type RNase superfamily (Beintema *et al*, 1997), is endowed with a strong cytotoxic activity against tumour cells *in vitro* (Laccetti *et al*, 1992) and is active against cancer cells *in vivo*, as well (Laccetti *et al*, 1994). Through protein engineering, a new dimeric RNase of human nature, HHP2-RNase, has been derived from human pancreatic RNase (HP-RNase). This was achieved by replacing five HP-RNase amino-acid residues (Q28, K31, K32, N34, E111) with the corresponding residues (L28, C31, C32, K34, G111) from BS-RNase. HHP2-RNase was even more powerful as a cytotoxic agent than BS-RNase, and as selective for malignant cells (Piccoli *et al*, 1999; Di Gaetano *et al*, 2001). This protein has the obvious advantage over BS-RNase of being little or not immunogenic at all for humans.

The exact mechanism of the antitumour action of RNases remains to be clarified. The binding of BS-RNase to a component of the extracellular matrix (Mastronicola *et al*, 1995) and of onconase to an unidentified cell surface receptor (Wu *et al*, 1993) is followed by endocytosis, translocation to the cytosol, degradation of RNA (rRNA or tRNA by BS-RNase and onconase, respectively) and suppression of protein synthesis leading to cell death. BS-RNase is internalised and enclosed in endosomes in both normal and malignant cells. Only in malignant cells, however, the RNase is transported into the trans Golgi network, whence it is released in the cytosol (Bracale *et al*, 2002). The dimeric structure of BS-RNase and HHP2-RNase is essential to enter the cytosol (Murthy *et al*, 1996; Antignani *et al*, 2001; Leland *et al*, 2001). The cytosolic ribonuclease inhibitor (cRI) tightly binds to internalised ribonucleases, thus blocking their cytotoxic activity; only ribonucleases that can evade cRI are capable of exerting a cytotoxic action. Also, the resistance of BS-RNase to cRI inhibition depends on the dimeric structure of the enzyme (Murthy *et al*, 1996; Antignani *et al*, 2001; Leland *et al*, 2001). On the other hand, onconase, the frog ribonuclease, is inhibited by frog but not mammalian cRI.

To verify whether the antineoplastic activity of ribonucleases was exerted on malignant cells independently from their specific genetic lesions, we performed a systematic study of the effects of BS-RNase and HHP2-RNase against genetically well-defined systems of thyroid tumour cells. Thyroid tumours are the most prevalent endocrine malignancy and some subtypes, anaplastic and poorly differentiated carcinomas, are associated with significant morbidity and mortality (Hedinger *et al*, 1989). Three different cell systems were used, derived from rat (Ambesi-Impiombato *et al*, 1980; Fusco *et al*, 1987; Portella *et al*, 1989) or human thyroid follicular cells (Kantor *et al*, 1978; Pang *et al*, 1989; Gioanni *et al*, 1991; Curcio *et al*, 1994 and carrying as diverse genetic alterations as a gain-of-function of Ki*-ras*, N*-ras*, *src*, *ret/ptc1* or B*-Raf* oncogenes or loss-of-function of p53. These cells display a full spectrum of progressive malignancy from normal differentiated, to immortalised, to oncogene-transformed, and to tumour- and metastasis-derived cells.

We show that HHP2-RNase and BS-RNase are highly cytotoxic for all tested tumour cell lines regardless of the specific genetic lesion carried by the target cells. Their activity was directly related to the degree of cell malignancy with no effects on the growth of normal cells. In stark contrast, doxorubicin showed its maximal cytotoxic effects against normal cells. A strong inhibitory activity was exerted by both RNases on the growth of tumours induced in nude mice, following subcutaneous (s.c.) injection of human thyroid carcinoma cells. The dimeric nature of the RNases scored essential for the antineoplastic effects, as no cytotoxic activity was exerted by monomeric RNases. These results suggest the potential utilisation of dimeric RNases for the therapy of aggressive human thyroid tumours, known to respond poorly to conventional chemotherapeutics.

## MATERIALS AND METHODS

### Cell lines

FRTL-5 is a normal, differentiated rat thyroid epithelial cell line (Ambesi-Impiombato *et al*, 1980). FRTL-5-*v-src* and FRTL-5-*v-*Ki*-ras* are *in vitro v-src*- and *v-*Ki*-ras-*transformed FRTL-5 cells (Fusco *et al*, 1987). TK-6 and MPTK-6 were derived from primary thyroid carcinomas and lung metastasis, respectively, induced in rats by the intrathyroid injection of the Kirsten Murine Sarcoma Virus (Portella *et al*, 1989). P5 is a primary culture of normal human differentiated thyrocytes (Curcio *et al*, 1994). HDF cells are normal human diploid fibroblasts (Kantor *et al*, 1978). The four human thyroid carcinoma-derived cell lines were Cal62 and ARO cells from anaplastic carcinomas (Pang *et al*, 1989; Gioanni *et al*, 1991) and NPA and TPC1 cells from papillary carcinomas (Pang *et al*, 1989; Ishizaka *et al*, 1990). Rat and human cell lines were cultured as previously reported (Kantor *et al*, 1978; Ambesi-Impiombato *et al*, 1980; Fusco *et al*, 1987; Pang *et al*, 1989; Portella *et al*, 1989; Gioanni *et al*, 1991; Curcio *et al*, 1994).

### Cell survival

At day 0, 100–500 cells (depending on the cell line) were plated. After 1 day, cells were treated for 24 h with increasing amounts of either ribonuclease (from 1.37 ng ml^−1^ to 13.7 *μ*g ml^−1^) or doxorubicin (from 29 pg ml^−1^ to 290 ng ml^−1^) corresponding to final concentrations for both drugs ranging from 0.05 to 500 nM. After 2–3 weeks, colonies were stained with crystal violet and counted, as previously described (Spalletti-Cernia *et al*, 1995).

### Gel electrophoresis of fragmented DNA

Agarose gel electrophoresis was performed as described by Hockenbery *et al* (1990). Briefly, DNA from control and treated cells (1 × 10^6^ cells) was extracted with phenol–chloroform, precipitated and subjected to electrophoresis on a 1.2% agarose gel. The agarose gel was stained with ethidium bromide and the resulting DNA fragmentation pattern was visualised by UV illumination.

### Fluorescence analysis of apoptosis

Cells were plated on coverglasses in 24-well plates and 24 h later, the RNase under test was added to a final concentration of 1.8. *μ*M. Following 72 h of treatment, cells were fixed in 3.7% formaldehyde in PBS for 15 min at room temperature. Cells were then permeabilised with 0.1% Triton X-100 for 5 min at room temperature and stained for 30 min with Hoechst 33258 (Sigma Chemical Co.) at a concentration of 0.5 *μ*g ml^−1^ in PBS. The stained cells were observed under an epifluorescent microscope (Axiovert 2, Zeiss) as previously reported (Piccoli *et al*, 1999).

### *In vivo* experiments

Nude mice (5 weeks old) were injected s.c. at day 0 with 1 × 10^6^ ARO cells (Pang *et al*, 1989). Mice were then treated with six injections in the peritumoral areas of 20 *μ*g g^−1^ of body weight of RNases. The first injection was given 24 h after the cells' implantation. The site of injection was marked. The subsequent administrations were repeated at 72 h intervals. During the period of treatment, tumour volume (*V*) was calculated by the following formula: *V*=width^2^ × length × 0.5 (Yanase *et al*, 1993). At day 22, all animals were killed and the tumours were excised and weighed.

Animal studies were conducted in accordance with the Italian regulation for experimentation on animals (Workman *et al*, 1998). All *in vivo* experiments were carried out with ethical committee approval and met the standards required by the UKCCCR guidelines. No mice showed signs of wasting or other signs of toxicity.

### Substances

BS-RNase was purified from bovine seminal vesicles as previously reported (D'Alessio *et al*, 2001). HHP2-RNase and HP-RNase were prepared and purified as described (Di Gaetano *et al*, 2001). Doxorubicin and RNase A were purchased from Sigma Chemical Co. (St Louis, MO, USA). All chemicals used were of reagent grade. The disposable materials used for cell culture were from Nunc (Mascia-Brunelli, Milano, Italy). Nude mice were purchased from Charles River Italia (Calco, Italy).

## RESULTS

### Selective cytotoxicity of BS-RNase and HHP2-RNase for thyroid cancer cells

The experiments reported in this paper were aimed at: (i) testing the possibility that the strength of the antineoplastic action of ribonucleases could be directly correlated with the degree of tumour cell malignancy; (ii) comparing the effects of RNases with those of doxorubicin, a conventional anticancer drug. This has been made possible by the use of rat and human cell systems, carrying well-characterised genetic lesions and displaying progressively increasing malignancy. All the used cell models refer to a specific epithelial tumour type, that is, carcinomas derived from thyroid epithelial follicular cells. The main biological properties of cell lines are summarised in Table 1

**Table 1:** Biological properties of the thyroid-derived cell lines used in this study and their sensitivity to doxorubicin and RNases

						**Antitumour agent IC_50_^c^**		
**Cell line**	**Genetic lesion**	**Doubling time (h)**	**CFE^a^**	**Tumour incidence^b^**	**Latency**	**Dox.**	**BS**	**HHP2**
*In vitro transformed rat cells*								
FRTL-5		33	0	0		1.5	>500	>500
FRTL-5-*v*-*src*	*v*-*src*	28	20	85	3 weeks	40	100	250
FRTL-5-*v*-Ki-*ras*	*v*-Ki-*ras*	14	49	100	5 days	200	10	18

*Tumour- and metastasis-derived rat cells*								
TK-6	*v*-Ki-*ras*	24	34	100	2.5 weeks	180	15	30
MPTK-6	*v*-Ki-*ras*	16	74	100	7–10 days	300	10	10

*Human cells*								
P5	—	58	0	0	—	0.9	>500	>500
HDF	—	36	0	0	—	2	>500	>500
TPC1	*ret/ptc1*	26	1	0	—	5	140	320
Cal 62	p53*; N-*ras*	24	20	100	7 days	Not done	74	37
NPA	p53−/−; B-raf*	13	82	100	20 days	300	5	5
ARO	p53*	10	92	100	4 days	280	60	61

a Colony-forming efficiency was measured in agar and calculated by the formula: (number of colonies formed/number of plated cells) × 100. Colonies larger than 64 cells were scored after 2–3 weeks.

b Tumorigenicity was assayed by injecting 10^6^ cells into either nude mice or syngenic rats. The incidence (%) was calculated as the per cent of animals with tumours over the total number of animals tested.

c The inhibiting concentration values (IC_50_) were determined by the colony-forming assay and are expressed as nM concentrations.

Values are means±s.d. of triplicate determinations from three independent experiments.

* Presence of a mutated protein.

, where they are subdivided into three different sets. The first one is represented by normal rat thyroid follicular FRTL-5 cells, which maintain the differentiated phenotype (Ambesi-Impiombato *et al*, 1980) and their *in vitro* transformed derivatives (FRTL-5 *v*-*src* and *v-*Ki*-ras*) obtained by retroviral transduction (Fusco *et al*, 1987). The second set is represented by TK-6 and MPTK-6, established in culture from primary thyroid carcinoma and lung metastasis, respectively, of rats subjected to intrathyroid injection of the *v-*Ki*-ras*-carrying retrovirus (Portella *et al*, 1989). The third set comprised normal human differentiated P5 thyrocytes (Curcio *et al*, 1994), normal human primary HDF fibroblasts, two papillary carcinoma-derived (TPC1 and NPA) and two anaplastic carcinoma-derived cell lines (Cal62 and ARO). The NPA cell line carries a double deletion of the p53 gene and an activating V599E point mutation in the B*-Raf* oncogene (Fagin *et al*, 1996; Kimura *et al*, 2003). Cal62 and ARO cells carry a mutated form of p53; the Cal62 cell line carries, in addition, a mutated N*-ras* gene (Namba *et al*, 1990; Fagin *et al*, 1993; Salvatore *et al*, 1996; Vecchio and Santoro, 2000). TPC1 cells carry a paracentric inversion of chromosome 10 causing the recombination of the tyrosine kinase-encoding domain of the *ret* receptor to the 5′-terminal region of the H4 gene, leading to the generation of the *ret/ptc1* oncogene (Ishizaka *et al*, 1990), a lesion commonly found in papillary carcinomas (Grieco *et al*, 1990).

We compared cell growth properties, colony-forming efficiency in soft agar and tumorigenicity upon s.c. injection in nude mice of the various cell lines; the results are reported in Table 1. Normal FRTL-5, P5 and HDF cells and thyroid papillary carcinoma-derived TPC1 cells did not show malignant features. TK-6 and FRTL-5-*v-src* cells showed an intermediate degree of malignancy, being able to grow in a semisolid medium and to induce tumours with long latency. MPTK-6, FRTL-5-*v-*Ki*-ras*, Cal 62, NPA and ARO cells displayed a highly malignant phenotype, as shown by their great efficiency to form colonies in soft agar and to induce tumours with short latency in nude mice (Table 1).

**Figure 1:**
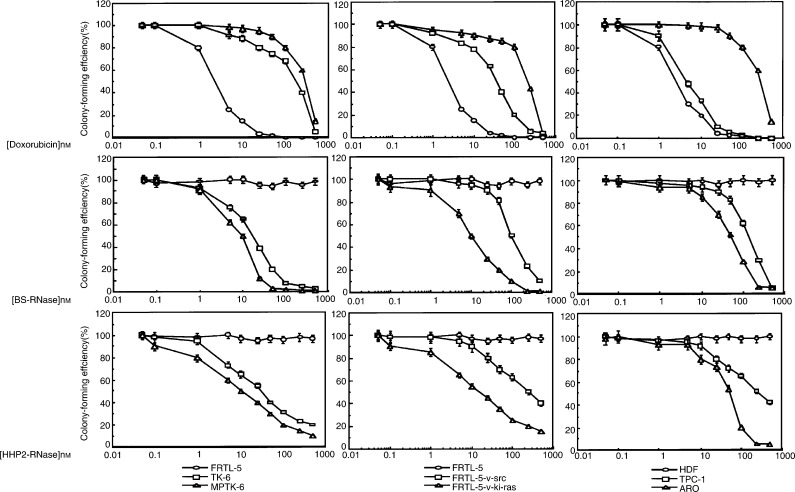
Cell survival of malignant and nonmalignant cell lines treated with the increasing concentration of doxorubicin, BS-RNase or HHP2-RNase. The average results±s.d. of three independent determinations. Cells were exposed to each molecule for 24 h. After 3 weeks, cell survival was measured by counting colony-forming units.

We tested the effects of BS-RNase and HHP2-RNase on all these cell lines in comparison to doxorubicin. Sensitivity to the compounds was expressed as IC_50_ (the concentration inhibiting 50% of the colony-forming efficiency) and is reported in Table 1. IC_50_ values were determined from the curves (Figure 1

) obtained by plotting the colony-forming efficiency *vs* the logarithm of drug molarity (the curves for P5, NPA and Cal62 are not shown). The results demonstrated a selective cytotoxicity of the two dimeric RNases towards cancer cells, the strength of the activity being directly related to the malignant phenotype of tumour cells. The most malignant cells (MPTK-6, FRTL-5*-v-*Ki*-ras*, NPA, Cal 62 and ARO) showed the lower values of IC_50_ (Table 1). No toxic effects were observed on normal counterparts of all cell systems. In contrast, an inverse relationship was observed between doxorubicin cytotoxicity and the degree of cell malignancy, the activity of doxorubicin being maximal against the normal cells (FRTL-5, HDF and P5 cells) (Table 1, Figure 1).

### BS-RNase and HHP2-RNase induce apoptosis in tumour cells

**Figure 2:**
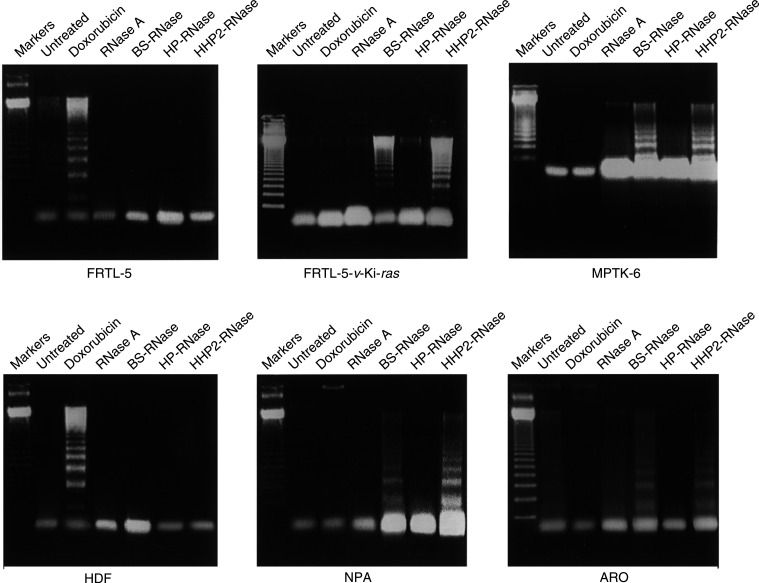
Analysis of DNA fragmentation in the malignant and nonmalignant cell lines treated with doxorubicin, BS-RNase or HHP2-RNase. Markers: molecular weight markers (123-bp multimers). These data are representative of at least three independent experiments.

We investigated the nature of cell death induced by treatment with RNases. Cells were analysed for the presence of fragmented DNA with the characteristic pattern of internucleosomal ladder, suggestive of apoptosis. As shown in Figure 2

, treatment with BS-RNase and HHP2-RNase, 72 h at 1.8 *μ*M (50 *μ*g ml^−1^), selectively promoted apoptosis in tumorigenic cells (MPTK-6, FRTL-5*-v-*Ki*-ras*, NPA and ARO cells), whereas apoptosis was undetected in the normal cell counterparts (FRTL-5 and HDF cells). Doxorubicin, instead (1.8 *μ*M, i.e. 1 *μ*g ml^−1^, for 24 h), promoted apoptosis in normal cells and not in tumour cells. After 72 h of treatment, doxorubicin killed all cells, malignant and nonmalignant (not shown). No proapoptotic activity was exerted by monomeric RNases (RNase A and HP-RNase) (Figure 2).

**Figure 3:**
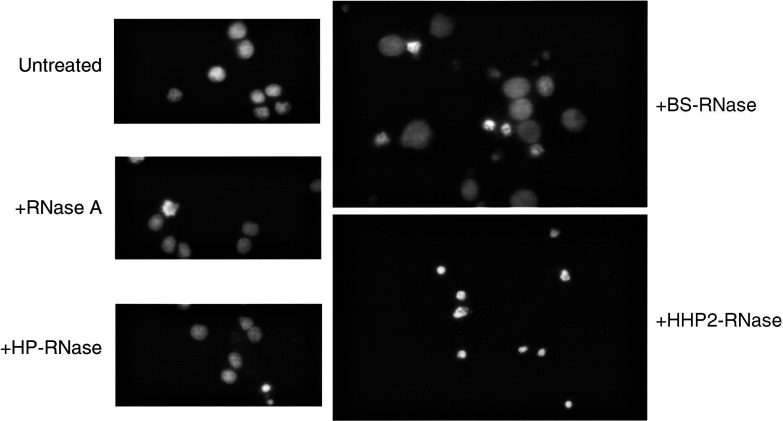
Fluorescence microscopy analysis of ARO cells treated with RNases. ARO cells were exposed for 72 h to the RNase under test (1.8 *μ*M). Cells were fixed and stained with Hoechst 33258 to reveal the nuclear signs of apoptosis and photographed (× 360).

The presence of apoptotic nuclear bodies was analysed by a fluorescence microscopy after staining with Hoechst 33258 (Figure 3

). In these experiments, we utilised the human cell line displaying the most malignant phenotype (ARO cell line). When this cell line was treated with either dimeric ribonuclease, it showed the characteristic chromatin condensation and margination and many fragmented nuclei. Again, monomeric RNase A and HP-RNase treatment did not cause any change in the nuclear morphology and number of cells (Figure 3). A quantitative estimation of BS-RNase- and HHP2-RNase-induced apoptosis gave values of 70±20 and 90±20%, respectively, of apoptotic cells. Similar results were obtained when the NPA cell line was used (data not shown).

### Antitumoral effects of BS-RNase and HHP2-RNase in nude mice

**Figure 4:**
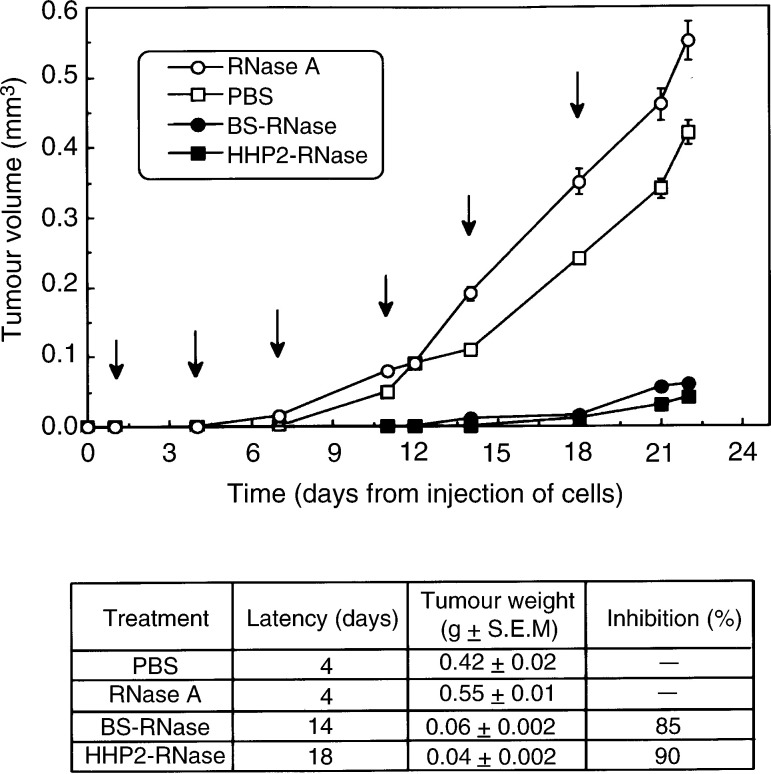
Time-dependent effect of BS-RNase (•-•) and HHP2-RNase (▪-▪) on tumour growth in mice inoculated s.c. (day 0) with 1 × 10^6^ ARO cells. RNases were administered in the peritumoral area six times at 72 h intervals as indicated by the arrows. Controls were treated with PBS (□-□) or RNase A (○-○). Latency periods and means of the weights of the tumours excised at the end of each treatment are reported in the table inset.

We utilised ARO cells, characterised by a very short latency period for tumour growth in mice, to assess the antineoplastic activity of ribonucleases *in vivo* in mice bearing human thyroid neoplastic cells. Nude mice, inoculated with 1 × 10^6^ ARO cells s.c., were treated with six injections in the peritumoral area of BS-RNase or HHP2-RNase (20 *μ*g g^−1^ of body weight). Administrations were repeated at 72 h intervals. A group of control mice was treated with pancreatic RNase A with the same protocol and dose; another control group was injected with PBS alone. As shown in Figure 4

, the antitumoral effects of both BS-RNase and HHP2-RNase were clearly evident during the whole period of mice treatment. In particular, the latency period for tumour appearance was highly prolonged in treated mice, reaching 14 and 18 days, respectively. At the end of the experiment, the average weight of the tumours from mice treated with BS-RNase and HHP2-RNase was 15 and 10% (85 and 90% of growth inhibition, respectively) with respect to the average weight of tumours grown in untreated mice or in mice treated with RNase A.

Finally, we sought to determine the toxicity of BS-RNase in healthy mice. Six injections intraperitoneal (i.p.) of 20 *μ*g g^−1^ of body weight of BS-RNase at 72 h intervals did not cause any effects on the survival of mice. The only side effect was a diminished body weight (20% reduction) with respect to untreated mice (data not shown).

## DISCUSSION

We have used a well-controlled thyroid cell model system to demonstrate specificity for malignant cells of the cytotoxic and proapoptotic effects exerted by two dimeric ribonucleases. The *in vitro* results were corroborated by a strong antitumour action of both RNases against tumours obtained by the injection of malignant cells in nude mice. Results from Phase II and III clinical trials in which onconase was used as the antitumour agent (Juan *et al*, 1998; Mikulski *et al*, 2002) support our belief that certain RNases exert a strong cytotoxicity towards cancer cells (Mikulski *et al*, 2002). However, onconase, at variance with the two ribonucleases that have been used in the present paper, has been shown to exert a cytotoxic effect also towards noncancerous cells and, at high concentrations, it leads to renal toxicity in mice (Haigis *et al*, 2003). The nature of cell death for both BS-RNase and HHP2-RNase has been found to be apoptotic. In fact, we have recently shown that BS-RNase and HHP2-RNase treatment triggers the activation of the execution caspase-3 (Spalletti-Cernia *et al*, 2003).

The availability of a genetically well-defined system of thyroid normal and tumour cells in our laboratory allowed us to perform a systematic study of the activities of BS-RNase and HHP2-RNase in comparison with the known chemotherapeutic drug doxorubicin. The cell systems used are representative of the different degrees of aggressiveness found in the various human thyroid tumours. Indeed, human thyroid carcinomas vary from the asymptomatic microcarcinoma to the differentiated carcinoma, to the anaplastic carcinomas, one of the most aggressive human cancers (Hedinger *et al*, 1989). Doxorubicin alone, and in combination with cisplatin, is the most widely used therapeutic agent for this last category of thyroid carcinomas; however, it does not improve survival in anaplastic carcinoma patients (Hedinger *et al*, 1989; Rosai *et al*, 1992). The findings reported here demonstrate that the strength of cytotoxic and apoptotic activity of RNases exerted on cells grown *in vitro* was directly related to the degree of cell malignancy. On the contrary, doxorubicin showed its maximal cytotoxic and apoptotic effect against normal cells.

Unlike doxorubicin, BS-RNase and HHP-2 RNase are not known to induce, nor do they appear to be influenced by, the multidrug-resistant phenotype, one of the most important mechanisms responsible for recurrences and metastatic invasion after chemotherapy. Indeed, MPTK-6 cells, highly susceptible to RNases, show the presence of a multidrug-resistant phenotype that is independent from the gp170 glycoprotein (Spalletti-Cernia *et al*, 1995).

Another important feature is that both RNases exert their cytotoxicity independent of the genetic lesions present in the target cancer cells. Here, we show that they exhibit a similar cytotoxic activity against cells bearing Ki-*ras* (FRTL5-*v-*Ki*-ras* and MPTK-6), N*-ras* (Cal62) and B*-raf* (NPA) mutations as well as against cells bearing p53 alterations. The most malignant cells, which are highly sensitive to both RNases, are representative of the whole possible spectrum of the p53 status. NPA cells show lack of any functional p53 (Fagin *et al*, 1996), ARO and Cal62 show a heterozygous mutant p53 (Fagin *et al*, 1993), whereas TK-6 and MPTK-6 have a normally functioning p53 (Vecchio *et al*, unpublished observations). Therefore, RNases cytotoxicity is also independent of the p53 status of the malignant cell line used. This has important therapeutic implications since it is well known that the majority of human cancers bear inactive p53 that renders tumour cells unable to undergo drug-induced apoptosis. In particular, mutations of p53 are the common feature of anaplastic thyroid cancer (Fagin *et al*, 1993). The *in vitro* results are strengthened by *in vivo* experiments in which peritumoral injection of both RNases inhibited the growth of human anaplastic thyroid cancer cells transplanted into nude mice and grown s.c. The protocol of RNases administration 1 day after the implantation of the tumour cells may seem to be of little applicability in the clinic. However, it must be considered that the growth rate of the tumour cells injected in mice is much higher than that in humans. In addition, nude mice injected with the ARO cells rapidly die if untreated and their treatment therefore must begin very early after implantation. For these reasons, 1 day after the s.c. implantation of the tumour cells in mice could well be considered equivalent to the initial phase of tumour growth in humans and could coincide with the beginning of a potential therapy. Moreover, RNase or other antineoplastic drug administration in the peritumoral area has been successfully used previously for the treatment of rat (Laccetti *et al*, 1994) or human thyroid tumour cells (Carlomagno *et al*, 2002; Barzon *et al*, 2002,^2003^). Obviously, the applicability of RNases in clinical situations and the site of administration of the antitumour molecules would depend largely on the type of the tumour, on its localisation and on the tumour growth rate. However, it is important to note that we have already shown that BS-RNase is capable of exerting a strong antitumour effect even when administered i.p. in a different *in vivo* experimental mouse system (Laccetti *et al*, 1994). Finally, it is important to underline that the peritumoral injection of RNase did not cause any adverse effect, such as death, body weight loss or changes in the physical appearance or behaviour of the treated mice. This is at variance with the slightly toxic effect (20% body weight reduction) observed with i.p. injections of BS-Rnase (see Results section). This further supports the choice of using peritumoral injections of the ribonucleases in the experimental system described here.

Furthermore, it must be considered that, in particular, human anaplastic thyroid tumours may represent a very suitable type of human tumour candidate for treatment with peritumoral or intratumoral injection of antineoplastic drugs. In fact, due to its late metastatic spreading, its severe local invasion and its relatively easy accessibility from the outside, this tumour lends itself in an ideal way to the peritumoral or intratumoral treatment such as the one described in this paper. Finally, it is important to consider that there are indeed already studies in the literature concerning clinical trials of human gliomas treated by using peritumoral or intratumoral injections of suicide and cytokine genes (Palù *et al*, 1999). It is interesting to note that a phase I clinical trial has been conducted by the same group for the treatment of human anaplastic thyroid carcinomas with peritumoral or intratumoral injections of the same genes (Palù, personal communication).

In conclusion, the dimeric RNases can be considered as drugs suitable for the development of novel alternative therapeutic agents against anaplastic thyroid tumours, to overcome the poor responsiveness of this type of tumour to conventional chemotherapeutic drugs, such as doxorubicin.
